# Development of a model for predicting gastrointestinal bleeding in patients with ischemic stroke after dual antiplatelet therapy

**DOI:** 10.3389/fneur.2025.1574278

**Published:** 2025-06-18

**Authors:** Yuncao Fan, Chunping Zhu, Jiamei Zhou, Renjie Yi, Jiaming Huang

**Affiliations:** ^1^Department of Cardiovascular, The First People’s Hospital of Wenling, Wenling, Zhejiang, China; ^2^Department of Gastroenterology, Ganzhou People’s Hospital, Ganzhou, Jiangxi, China

**Keywords:** ischemic stroke, dual antiplatelet therapy, gastrointestinal bleeding, risk factor, model

## Abstract

**Objective:**

To establish a model for predicting gastrointestinal bleeding in patients with ischemic stroke after dual antiplatelet therapy (DAPT).

**Methods:**

A model for predicting gastrointestinal bleeding in patients with ischemic stroke after DAPT was established based on a retrospective study that involved 1,217 patients diagnosed with ischemic stroke in the Neurology Department of Nanchang University Affiliated Ganzhou Hospital from January 2019 to June 2021. A receiver operating characteristic curve was constructed to evaluate the model’s power. Data from patients with ischemic stroke between July and December 2021 were used to validate the power of the model.

**Results:**

A total of 1,217 patients with ischemic stroke between January 2019 and June 2021 were included in the model. The cohort comprised 1,164 patients in the non-gastrointestinal bleeding group and 53 in the gastrointestinal bleeding group. Multivariate logistic regression analysis revealed that age, fibrinogen level, neutrophil-to-lymphocyte ratio, and National Institute of Health Stroke Scale score were independent risk factors for gastrointestinal bleeding. A model for predicting gastrointestinal bleeding in patients with ischemic stroke after DAPT was established, Logit(P) = −7.269 + 0.074 ×1 + 0.071 ×2 + 0.361 ×3 + 0.082 ×4 (X1, National Institute of Health Stroke Scale score; X2, neutrophil-to-lymphocyte ratio; X3, fibrinogen; X4, activated partial thromboplastin time). Receiver operating characteristic analysis showed that the area under the curve for the model was 0.733. Data from the validation group showed that the area under the curve for the model was 0.665.

**Conclusion:**

A model for predicting gastrointestinal bleeding in patients with ischemic stroke after DAPT was established and demonstrated its predictive ability. Although the predictive ability of the model was not perfect, this was an important attempt. Further studies are needed to establish better models to predict gastrointestinal bleeding.

## 1 Introduction

Ischemic stroke is a serious neurological dysfunction caused by insufficient cerebral blood supply. It is a leading cause of death and disability worldwide (1). In 2021, there were 11.9 million incident strokes globally, and 65.3% of which were ischemic (2). Dual antiplatelet therapy (DAPT) with aspirin and clopidogrel is effective in reducing recurrent ischemic stroke, but increases the risk of gastrointestinal bleeding. Risk factors for gastrointestinal bleeding in patients with ischemic stroke after DAPT includes stroke severity and neutrophil-to-lymphocyte ratio (NLR) (3, 4). Because gastrointestinal bleeding is a risk factor for increased mortality in acute cerebral infarction (5), predicting its occurrence in patients with ischemic stroke after DAPT is crucial. However, a predictive model for this specific population is currently lacking. Therefore, we retrospectively analyzed the data of patients with ischemic stroke receiving DAPT at the Nanchang University Affiliated Ganzhou Hospital to develop a model for predicting gastrointestinal bleeding.

## 2 Materials and methods

### 2.1 Study design and patients

This retrospective clinical study was approved by the Ethics Committee of the Nanchang University Affiliated Ganzhou Hospital and conducted in accordance with the Declaration of Helsinki. Consent was obtained from all patients.

Data from patients with ischemic stroke admitted to the Neurology Department between January 2019 and June 2021 were collected to build a model for predicting gastrointestinal bleeding in patients with ischemic stroke after DAPT. Data from patients with ischemic stroke admitted between July and December 2021 were collected to validate the model. Ischemic stroke was defined according to the World Health Organization clinical criteria as the sudden onset of neurological deficits (6).

Inclusion criteria were: (a) patients with ischemic stroke who were confirmed by computed tomography and/or magnetic resonance imaging, (b) hospitalization within 1 week symptom onset, and (c) patients who had dual antiplatelet therapy with aspirin and clopidogrel for more than 12 h. The exclusion criteria were as follows: (a) patients who had cerebral hemorrhage and pulmonary tuberculosis; (b) patients who had gastrointestinal bleeding symptoms, such as hematemesis or melena, in less than 12 h of receiving aspirin and clopidogrel; and (c) patients whose data were incomplete.

### 2.2 Patient classification

The patients were divided into bleeding and non-bleeding groups, defined by the presence or absence of in-hospital gastrointestinal bleeding.

In-hospital gastrointestinal bleeding was defined as fresh blood or coffee ground emesis, hematemesis, melena, hematochezia, or a positive fecal occult blood test during hospitalization (7).

### 2.3 Data collection

Data from all patients were collected from the electronic medical record system of our hospital. The collected data included demographics, personal history, previous history, treatment, laboratory tests, ischemia site, and National Institute of Health Stroke Scale (NIHSS) score, which was assessed by physicians at admission. Patient demographics included age and sex. Personal histories included smoking and drinking habits. Patients with hypertension, coronary heart disease, diabetes, cerebral infarction, cerebral hemorrhage, gout, peptic ulcer, and chronic kidney disease were included in the study. Treatments included cerebral arteriography, mechanical ventilation, intravenous thrombolysis, urokallikrein, and heparin. Laboratory tests performed within 24 h of admission included white blood cell count, neutrophils, lymphocytes, platelets, mean platelet volume, nitrogen, creatinine, prothrombin time, fibrinogen, activated partial thromboplastin time (APTT), D-dimer, total bilirubin, and albumin. The NLR, which is regarded as an inflammatory biomarker, is the result of the neutrophil count divided by the lymphocyte count.

On day 1, after admission, all patients received a loading dose of 300 mg of aspirin and 300 mg of clopidogrel. Subsequently, 100 mg of aspirin and 75 mg of clopidogrel per day were administered.

### 2.4 Statistical analysis

Statistical analyses were performed using SPSS software (version 22, Co., Ltd., Tokyo, Japan). The collected data included continuous and categorical variables. Continuous variables, which are represented by mean ± standard deviation, were analyzed using the Student’s t-test or the rank sum test. Categorical variables are represented by frequency and percentage and were analyzed using the chi-square test.

Univariate logistic regression was used to select related factors, and factors with *p*-value < 0.1 were included in the multivariate logistic regression. A model for predicting gastrointestinal bleeding after DAPT in patients with ischemic stroke was established based on multivariate logistic regression results. The sensitivity and specificity of the model were determined and validated by receiver operating characteristic (ROC) curves. *p* < 0.05 was regarded as significant statistically.

## 3 Results

### 3.1 Basic characteristics of patients

A total of 1,217 ischemic strokes between January 2019 and June 2021 were included to construct the model. There were 1,164 patients in the non-gastrointestinal bleeding group, of whom 839 were male and 690 were older than 60 years. There were 53 patients in the gastrointestinal bleeding group, 46 of whom were male, and 32 were older than 60 years (Table 1).

**Table 1:** Baseline characteristics of patients in the model group.

Factors	Non-gastrointestinal bleeding group (*n* = 1,164)	Gastrointestinal bleeding group (*n* = 53)	*p*-value
Sex (male)	839(72.08%)	46(86.79%)	0.019
Age(≥60 years)	690(59.28%)	32 (60.38%)	0.873
Cerebral angiography	165 (14.18%)	10 (18.87%)	0.341
Mechanical ventilation	16 (1.37%)	3 (5.66%)	0.014
Thrombolysis	57 (4.90%)	1 (1.89%)	0.314
Kallidinogenase	255 (21.91%)	7 (13.21%)	0.132
Heparin	115 (13.31%)	8 (15.09%)	0.218
Hypertension	757 (65.03%)	30 (56.60%)	0.209
CHD	53 (4.55%)	3 (5.66%)	0.707
Diabetes	229 (19.67%)	6 (11.32%)	0.132
Previous history of cerebral infarction	229 (19.67%)	8 (15.09%)	0.410
Gout	22 (1.89%)	3 (5.66%)	0.058
Peptic ulcer	19 (1.63%)	2 (3.77%)	0.242
COPD	10 (0.86%)	0 (0.00%)	0.498
CKD	4 (0.34%)	0 (0.00%)	0.669
Smoking	420 (36.08%)	25 (47.17%)	0.101
Drinking	191 (16.41%)	5 (9.43%)	0.177

A total of 333 patients with ischemic stroke between June and December 2021 were included to validate the model. There were 315 patients in the non-gastrointestinal bleeding group, 213 of whom were male, and 196 were older than 60 years. There were 18 patients in the gastrointestinal bleeding group, 15 of whom were male, and 13 were older than 60 years (Table 2).

**Table 2:** Baseline characteristics of patients in the validation group.

Factors	Non-gastrointestinal bleeding group (*n* = 315)	Gastrointestinal bleeding group (*n* = 18)	*p*-value
Sex (male)	213 (67.62%)	15 (83.33%)	0.163
Age(≥60 years)	196 (62.22%)	13 (72.22%)	0.393
Cerebral angiography	73 (23.17%)	7 (38.89%)	0.129
Mechanical ventilation	0 (0.00%)	1 (5.56%)	0.000
Thrombolysis	9 (2.86%)	1 (5.56%)	0.514
Kallidinogenase	115 (36.51%)	11 (61.11%)	0.036
heparin	18 (5.71%)	2 (11.11%)	0.349
Hypertension	215 (68.25%)	10 (55.56%)	0.263
CHD	18 (5.71%)	2 (11.11%)	0.349
Diabetes	74 (23.49%)	3 (16.67%)	0.504
Previous history of cerebral infarction	68 (21.59%)	2 (11.11%)	0.289
Gout	6 (1.90%)	0 (0.00%)	0.555
Peptic ulcer	4 (1.27%)	0 (0.00%)	0.631
COPD	1 (0.32%)	1 (5.56%)	0.005
CKD	4 (1.27%)	0 (0.00%)	0.631
Smoking	110 (34.92%)	7 (38.89%)	0.732
Drinking	49 (15.56%)	0 (0.00%)	0.070

### 3.2 Comparison of basic and laboratory tests of patients in the model group

There were more male patients in the gastrointestinal bleeding group than in the non-gastrointestinal bleeding group, and more patients in the gastrointestinal bleeding group required mechanical ventilation. White blood cell count, total bilirubin, fibrinogen, APTT, NLR, and NIHSS were higher in the gastrointestinal bleeding group than in the non-gastrointestinal bleeding group (Tables 1, 3).

**Table 3:** Laboratory tests of patients in the model group.

Factors	Non-gastrointestinal bleeding group (*n* = 1,164)	Gastrointestinal bleeding group (*n* = 53)	*p*-value
WBC(10^9^/L)	7.93 ± 2.80	9.3891 ± 3.33011	0.000
Red blood cell distribution width (%)	13.11 ± 1.33	13.46 ± 2.00	0.067
Platelet(10^9^/L)	252.03 ± 72.67	248.43 ± 77.88	0.726
Platelet mean volume (fL)	10.05 ± 1.22	10.1647 ± 1.17	0.507
Total bilirubin (μmol/L)	15.37 ± 7.39	17.80 ± 10.35	0.025
Albumin (g/L)	40.30 ± 4.39	39.14 ± 5.36	0.068
Nitrogen (mmol/L)	5.02 ± 2.17	5.61 ± 2.54	0.057
Creatinine (μmol/L)	79.43 ± 40.50	86.044 ± 32.60	0.246
PT (s)	10.96 ± 0.93	11.16 ± 1.86	0.150
Fibrinogen (g/L)	2.92 ± 0.84	3.41 ± 1.22	0.000
APTT (s)	26.68 ± 3.32	28.08 ± 4.00	0.003
D-dimer (μg/L)	0.73 ± 2.26	0.98 ± 1.76	0.421
NLR	4.00 ± 3.87	7.39 ± 7.72	0.000
NIHSS	4.73 ± 4.35	7.91 ± 7.40	0.000

### 3.3 Univariate logistic regression selecting factors associated with gastrointestinal bleeding

Univariate logistic regression showed that sex, mechanical ventilation, gout, white blood cell count, red blood cell width distribution, total bilirubin, albumin, nitrogen, fibrinogen, APTT, NLR, and NIHSS were associated with gastrointestinal bleeding (*p* < 0.1) (Table 4). All these factors were included in the multivariate logistic regression analysis.

**Table 4:** Uni-variate logistic regression selecting factors associated with gastrointestinal bleeding.

Factors	β	OR	95% CI for OR	*p*-value
Sex (male)	−0.934	0.393	0.176, 0.879	0.023
Mechanical ventilation	−1.46	0.232	0.066, 0.823	0.024
Gout	−1.136	0.321	0.093, 1.108	0.072
NIHSS	0.102	1.107	1.061, 1.155	0.000
WBC	0.131	1.140	1.060, 1.226	0.000
Red blood cell distribution width	0.140	1.150	0.988, 1.339	0.071
Total bilirubin	0.031	1.032	1.004, 1.061	0.027
Albumin	−0.054	0.948	0.895, 1.004	0.066
Nitrogen	0.073	1.075	0.993, 1.165	0.074
Fibrinogen	0.516	1.676	1.294, 2.172	0.000
APTT	0.113	1.120	1.040, 1.206	0.003
NLR	0.093	1.097	1.055, 1.141	0.000

### 3.4 Independent risk factors for gastrointestinal bleeding in patients with ischemic stroke after DAPT

Multivariate logistic regression analysis was used to select variables with a *p* < 0.1 in univariate logistic regression analysis. The result showed that NIHSS, NLR, fibrinogen, and APTT were independent risk factors for gastrointestinal bleeding.

### 3.5 A model for predicting gastrointestinal bleeding in patients with ischemic stroke after DAPT

Based on the results of multivariate logistic regression analysis, a model for predicting gastrointestinal bleeding in patients with ischemic stroke after DAPT was established: logit (P) = −7.269 + 0.074 ×1 + 0.071 ×2 + 0.361 ×3 + 0.082 ×4 (X1, NIHSS; X2, NLR; X3, fibrinogen; X4, APTT) (Table 5).

**Table 5:** Multi-variate logistic regression selecting factors associated with gastrointestinal bleeding.

Factors	β	OR	95% CI for OR	*p*-value
NIHSS	0.074	1.076	1.027, 1.128	0.002
Fibrinogen	0.361	1.435	1.101, 1.869	0.007
APTT	0.082	1.085	1.001, 1.176	0.046
NLR	0.071	1.073	1.032, 1.117	0.000
Constant	−7.269	0.001		0.000

### 3.6 Discriminative power and validation of the model

**Figure 1:**
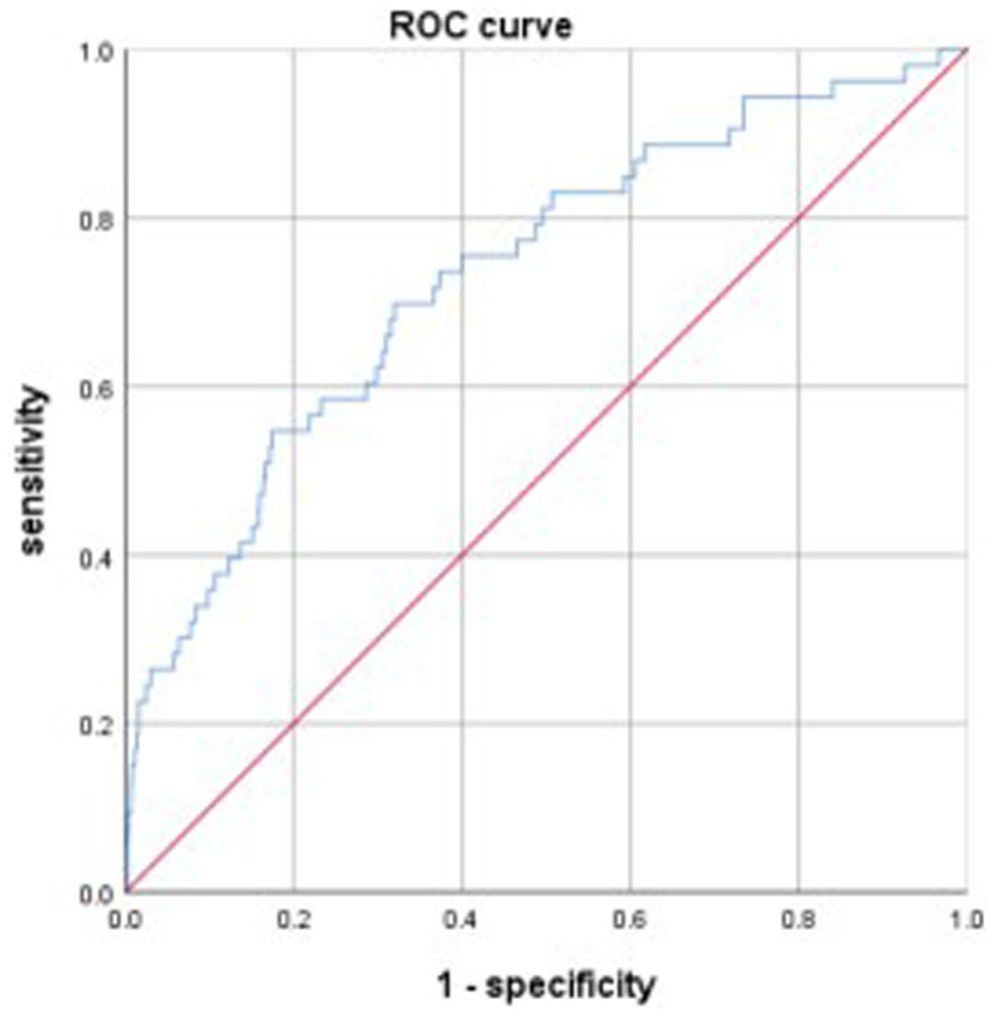
ROC curve reflecting the power of the model by model group.

**Figure 2:**
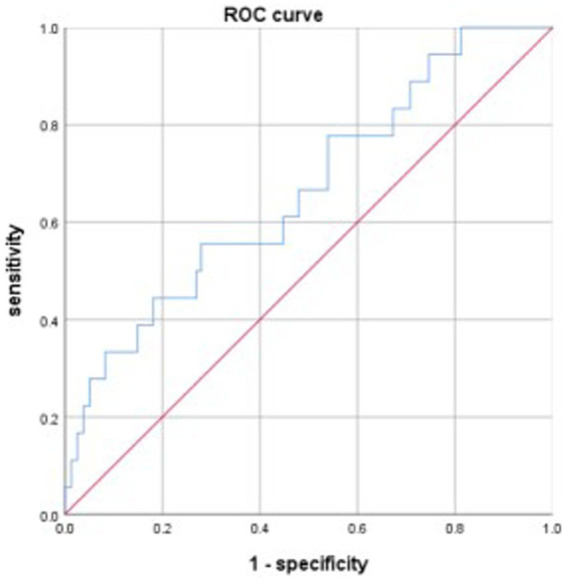
ROC curve reflecting the power of the model by validation group.

The discriminative power of the model was tested using a ROC curve analysis. The area under the curve (AUC) of the model was 0.733, the maximum Youden index was 0.378, with an optimal cut-off value of −3.257, and the sensitivity and specificity were 0.698 and 0.68, respectively (Figure 1). Data from patients treated between July and December 2021 were used to validate the model, and the AUC of the model was 0.665 (Figure 2).

## 4 Discussion

Several guidelines recommend that patients with acute ischemic stroke take dual antiplatelet therapy to prevent recurrent ischemic stroke (8–10). DAPT is more effective than single antiplatelet therapy in preventing ischemic stroke recurrence, but it carries an increased risk of gastrointestinal bleeding. Few studies have studied the risk factors for gastrointestinal bleeding in patients with ischemic stroke receiving DAPT (3, 4). No model has been developed to predict gastrointestinal bleeding after DAPT in patients with ischemic stroke.

In this study, through multivariate logistic regression analysis identified four independent risk factors for gastrointestinal bleeding: age, NIHSS score, fibrinogen, and NLR. Although sex, mechanical ventilation, total bilirubin level, and APTT showed significant differences, multivariate analysis revealed that they were not independent risk factors for gastrointestinal bleeding.

NIHSS score is widely used to assess ischemic stroke severity in clinical settings. Higher scores indicate greater severity. Several studies have found that NIHSS score is a risk factor for gastrointestinal bleeding in patients with ischemic stroke (11, 12). Patients with higher NIHSS scores have a substantially increased risk of developing gastrointestinal bleeding after DAPT which might be one of the reasons why the European Stroke Organization recommends DAPT only for patients with minor stroke and high-risk transient ischemic attack (8).

The NLR, the ratio of neutrophils to lymphocytes, is considered a biomarker of inflammation. Inflammation plays an important role in the physiological mechanisms of acute ischemic stroke (13). The infiltration of neutrophils and lymphocytes leads to the release of neurotoxins and cytokines, further impairing the neurological function (14, 15). Many studies have demonstrated that a higher NLR is closely linked to a poor prognosis of ischemic stroke (16, 17). A study found that in patients with acute ischemic stroke, the NLR was not only associated with early post-thrombolysis neurological deterioration but also with early post-thrombolysis neurological improvement (18). In this study, we also found that NLR was a risk factor for gastrointestinal bleeding in ischemic stroke after DAPT.

This study developed a model to predict gastrointestinal bleeding in patients with ischemic stroke after DAPT, Logit(P) = −7.269 + 0.074 ×1 + 0.071 ×2 + 0.361 ×3 + 0.082 ×4 (X1, NIHSS score; X2, NLR; X3, fibrinogen; X4, APTT). The AUC of the model for predicting gastrointestinal bleeding was 0.733, with a sensitivity of 0.698 and specificity of 0.68. Data from the validation group showed that the AUC for the model was 0.665. Based on the AUC, this is not an excellent model; however, it represents a meaningful attempt to predict gastrointestinal bleeding in patients with ischemic stroke after DAPT. This attempt may encourage more further research to develop better models for predicting gastrointestinal bleeding in patients with ischemic stroke after DAPT, ultimately benefiting patients.

This study has several limitations. First, the nature of this single-center retrospective study restricts its reliability. Second, gastrointestinal bleeding group had a relatively small sample size. Third, although the model was validated in an external cohort, its predictive ability was poor. Fourth, there were a few patients who accepted Plavix resistance test, so, Plavix resistance results were not included in the study. Fifth, prior aspirin use was not included in the study, which might be a confounding factor of gastrointestinal bleeding. Further research is needed to address these limitations.

In conclusion, this study developed and validated a model to predict gastrointestinal bleeding in patients with ischemic stroke after DAPT. Although the model’s predictive ability requires improvement, it represents a valuable contribution. Although the model’s predictive ability requires improvement, it represents a valuable contribution Further research is needed to establish a better model for predicting gastrointestinal bleeding.

## Data Availability

The original contributions presented in the study are included in the article/supplementary material, further inquiries can be directed to the corresponding author.

## References

[1] Wafa HA Marshall I Wolfe CDA Xie W Johnson CO Veltkamp R. Burden of intracerebral haemorrhage in Europe: forecasting incidence and mortality between 2019 and 2050. Lancet Reg Health Eur. (2024) 38:100842. doi: 10.1016/j.lanepe.2024.100842, PMID: 38362494 PMC10867656

[2] GBD. Stroke risk factor collaborators. Global, regional, and national burden of stroke and its risk factors, 1990–2021: a systematic analysis for the Global Burden of Disease Study 2021. Lancet Neurol. (2024) 23:973–1003. doi: 10.1016/S1474-4422(24)00369-739304265

[3] Huang J Liao F Tang J Shu X. Risk factors for gastrointestinal bleeding in patients with cerebral infarction after dual antiplatelet therapy. Clin Neurol Neurosurg. (2023) 231:107802. doi: 10.1016/j.clineuro.2023.107802, PMID: 37295199

[4] Huang J Liao F Luo Y Shu X. Neutrophil-to-lymphocyte ratio at admission is a risk factor for in-hospital gastrointestinal bleeding in acute ischemic stroke patients after dual antiplatelet therapy: a case control study. J Stroke Cerebrovasc Dis. (2023) 32:107325. doi: 10.1016/j.jstrokecerebrovasdis.2023.107325, PMID: 37660552

[5] Huang ZX Gu HQ Yang X Wang CJ Wang YJ Li ZX. Risk factors for in-hospital mortality among acute ischemic stroke patients in China: a nationwide prospective study. Neurol Res. (2021) 43:387–95. doi: 10.1080/01616412.2020.1866356, PMID: 33357098

[6] Aho K Harmsen P Hatano S Marquardsen J Smirnov VE Strasser T. Cerebrovascular disease in the community: results of a WHO collaborative study. Bull World Health Organ. (1980) 58:113–30. PMID: 6966542 PMC2395897

[7] Davenport RJ Dennis MS Warlow CP. Gastrointestinal hemorrhage after acute stroke. Stroke. (1996) 27:421–4. doi: 10.1161/01.str.27.3.421 8610306

[8] Dawson J Merwick Á Webb A Dennis M Ferrari J Fonseca AC. For the European stroke organisation European stroke organisation expedited recommendation for the use of short-term dual antiplatelet therapy early after minor stroke and high-risk TIA. Eur Stroke J. (2021) 6:CLXXXVII–CXCI. doi: 10.1177/23969873211000877, PMID: 34414300 PMC8370083

[9] Kleindorfer DO Towfighi A Chaturvedi S Cockroft KM Gutierrez J Lombardi-Hill D. 2021 guideline for the prevention of stroke in patients with stroke and transient ischemic attack: a guideline from the American Heart Association/American Stroke Association. Stroke. (2021) 52:e364–467. doi: 10.1161/STR.0000000000000375, PMID: 34024117

[10] Powers WJ Rabinstein AA Ackerson T Adeoye OM Bambakidis NC Becker K. Guidelines for the early management of patients with acute ischemic stroke: 2019 update to the 2018 guidelines for the early Management of Acute ischemic stroke: a guideline for healthcare professionals from the American Heart Association/American Stroke Association. Stroke. (2019) 50:e344–418. doi: 10.1161/STR.0000000000000211 31662037

[11] Wang T Zhu D Kong L Mu C Li C Hu L. Effect of upper gastrointestinal bleeding on prognosis of middle-aged patients with acute ischemic stroke: a retrospective study. Ann Palliat Med. (2021) 10:5494–501. doi: 10.21037/apm-21-907, PMID: 34044566

[12] Fu J. Factors affecting the occurrence of gastrointestinal bleeding in acute ischemic stroke patients. Med. (2019) 98:e16312. doi: 10.1097/MD.0000000000016312, PMID: 31305417 PMC6641696

[13] Shan Y Zhang R Lu J Huang L Wang Y Long F. Neutrophil to lymphocyte ratio and five-year mortality in patients with acute ischemic stroke. Heliyon. (2024) 10:e36827. doi: 10.1016/j.heliyon.2024.e36827, PMID: 39281440 PMC11395762

[14] Shekhar S Cunningham MW Pabbidi MR Wang S Booz GW Fan F. Targeting vascular inflammation in ischemic stroke: recent developments on novel immunomodulatory approaches. Eur J Pharmacol. (2018) 833:531–44. doi: 10.1016/j.ejphar.2018.06.028, PMID: 29935175 PMC6090562

[15] Kim JY Park J Chang JY Kim SH Lee JE. Inflammation after ischemic stroke: the role of leukocytes and glial cells. Exp Neurobiol. (2016) 25:241–51. doi: 10.5607/en.2016.25.5.241, PMID: 27790058 PMC5081470

[16] Jayaraj RL Azimullah S Beiram R Jalal FY Rosenberg GA. Neuroinflammation: friend and foe for ischemic stroke. J Neuroinflammation. (2019) 16:142. doi: 10.1186/s12974-019-1516-2, PMID: 31291966 PMC6617684

[17] Yuan M Han B Xia Y Liu Y Wang C Zhang C. Augmentation of peripheral lymphocyte-derived cholinergic activity in patients with acute ischemic stroke. BMC Neurol. (2019) 19:236. doi: 10.1186/s12883-019-1481-5, PMID: 31615442 PMC6792255

[18] Gong P Liu Y Gong Y Chen G Zhang X Wang S. The association of neutrophil to lymphocyte ratio, platelet to lymphocyte ratio, and lymphocyte to monocyte ratio with post-thrombolysis early neurological outcomes in patients with acute ischemic stroke. J Neuroinflammation. (2021) 18:51. doi: 10.1186/s12974-021-02090-6, PMID: 33610168 PMC7896410

